# Isolation and Identification of Secondary Metabolites Produced by Phytopathogenic Fungus *Corynespora cassiicola* from *Hevea brasiliensis*

**DOI:** 10.3390/molecules27217360

**Published:** 2022-10-29

**Authors:** Xiaoyan Yang, Zhikai Guo, Yang Yang, Ailiman Abulaizi, Zijun Xiong, Shiqing Zhang, Boxun Li, Guixiu Huang

**Affiliations:** 1 State Key Laboratory Breeding Base of Green Pesticide and Agricultural Bioengineering, Key Laboratory of Green Pesticide and Agricultural Bioengineering, Ministry of Education, Guizhou University, Guiyang 550025, China; 2 Hainan Key Laboratory of Tropical Microbe Resources, Institute of Tropical Bioscience and Biotechnology, Chinese Academy of Tropical Agricultural Sciences & Key Laboratory for Biology and Genetic Resources of Tropical Crops of Hainan Province, Hainan Institute for Tropical Agricultural Resources, Haikou 571101, China; 3 Key Laboratory of Integrated Pest Management on Tropical Crops, Ministry of Agriculture and Rural Affairs & Hainan Key Laboratory for Monitoring and Control of Tropical Agricultural Pests, Environment and Plant Protection Institute, Chinese Academy of Tropical Agricultural Sciences, Haikou 571101, China; 4 State Key Laboratory of Pharmaceutical Biotechnology, Institute of Functional Biomolecules, School of Life Sciences, Nanjing University, Nanjing 210023, China

**Keywords:** *Hevea brasiliensis*, phytopathogenic fungi, *Corynespora cassiicola*, Corynesporascaceae, secondary metabolites, phytotoxicity

## Abstract

The secondary metabolites of the phytopathogenic fungus *Corynespora cassiicola* CC01 from *Hevea brasiliensis* were investigated. As a result, two new compounds, 5-acetyl-7-hydroxy-6- methoxybenzofuran-2(3*H*)-one (**1**) and (*S*)-2-(2,3-dihydrofuro [3,2-*c*]pyridin-2-yl)propan-2-ol (**2**), together with seven known compounds, 4,6,8-trihydroxy-3,4-dihydronaphthalen-1(2*H*)-one (**3**), 3,6,8-trihydroxy-3,4-dihydronaphthalen-1(2*H*)-one (**4**), curvulin acid (**5**), 2-methyl-5-carboxymethyl- 7-hydroxychromone (**6**), tyrosol (**7**), *p*-hydroxybenzoic acid (**8**) and cerevisterol (**9**), were isolated from the fermentation extract by comprehensive silica gel, reverse phase silica gel, Sephadex-LH20 column chromatography and high-performance liquid chromatography (HPLC). The structures of these compounds were identified by using high-resolution electrospray mass spectrometry (HRESIMS), nuclear magnetic resonance spectroscopy (NMR), optical rotation, ultraviolet and infrared spectroscopy techniques and a comparison of NMR data with those reported in the literature. Compounds **1** and **2** were new compounds, and compounds **3**–**9** were discovered from this phytopathogenic fungus for the first time. Compounds **1**–**9** were tested for phytotoxicity against the fresh tender leaf of *Hevea brasiliensis*, and the results show that none of them were phytotoxic. Additionally, these compounds were subjected to an antimicrobial assay against three bacteria (*E. coli*, *methicillin-resistant Staphylococcus aureus* and *Micrococcus luteus*), but they showed no activity.

## 1. Introduction

Natural rubber is both an important industrial raw material and a strategic material [1]. For a long time, rubber leaf diseases threatened the safety of rubber production. Rubber tree *Corynespora* leaf fall (CLF) disease, caused by the fungus *Corynespora cassiicola*, is a devastating rubber tree leaf disease in many countries in Asia and Africa, and also threatens rubber production in China [2]. Cassiicolin, a small 27 amino acid glycoprotein with six cysteines engaged in three disulphide bonds, is the only phytotoxin characterised in *C. cassiicola*. Based on the amino acid residues and the sequence of the cassicolin-encoding gene, there are six different toxin classes (Cas1–6). Some strains lack the cassicolin-encoding gene, called Cas0 isolates, which can also infect *Hevea brasiliensis* and other host plants, indicating *C. cassiicola* can produce other phytotoxic compounds [3]. At present, abundant phytotoxic secondary metabolites, such as polyketides, peptides, terpenoids and nitrogenous metabolites, were isolated from many plant pathogenic fungi and have played an important role in pathogenicity [4,5]. For example, botryoisocoumarinn A and neoisocoumarin, previously isolated from *Neufusicoccum batangarum*, induced necrotic lesions around the inoculation points in a host (cactus pear) [6]. Both nectriapyrone and methylphomapyrone C were toxic to a number of non-host plants, according to the leaf puncture assay [7]. Polanrazines A, B, C, D and E, isolated from Polish isolates of *Leptosphaeria maculans*, showed moderate toxicity on leaves of brown mustard [8]. In addition, some secondary metabolites were isolated from *C. cassiicola*. Herbarin is a naphthoquinone congener, previously isolated from *Corynespora* sp. BA-10763 [9]. Twelve new chromone derivatives, corynechromones A−L, were previously reported from the sponge-derived fungus *C. cassiicola* XS-200900I7, collected from the Xisha Islands coral reef in the South China Sea [10]. Additionally, coryoctalactones A–E were isolated from the endolichenic fungal strain *C. cassiicola* (JCM 23.3). All these compounds were evaluated for their antimicrobial, cytotoxic and antitrypanosomal activities. Unfortunately, none of these isolated natural products proved to be active in any of the bioassays performed [11]. These previous results revealed that the *C. cassiicola* species had the potential to produce structurally diverse metabolites.

In our previous study, we analyzed the population and pathogenicity diversity of *C. cassiicola* collected from tropical plants in China and found the Cas5 and Cas2 isolates could cause CLF disease in rubber trees in China, with Cas5 isolates being the dominant strains [12]. We further determined gapless genome sequences of Cas5 (strain CC01) and Cas2 (strain YN49) isolates and found *C. cassiicola* contained abundant secondary metabolite gene clusters, and these clusters significantly differed among the different Cas types and exhibited a different virulence. These data indicate that the phytogenic *C. cassiicola* can synthesize abundant secondary metabolites and may contain important pathogenic factors [13]. In order to uncover the secondary metabolites and phytotoxic metabolites produced by *C. cassiicola*, we investigated the fermentation extract of CC01 strain, a representative strain of Cas5 isolates in this study.

## 2. Results

**Figure 1:**
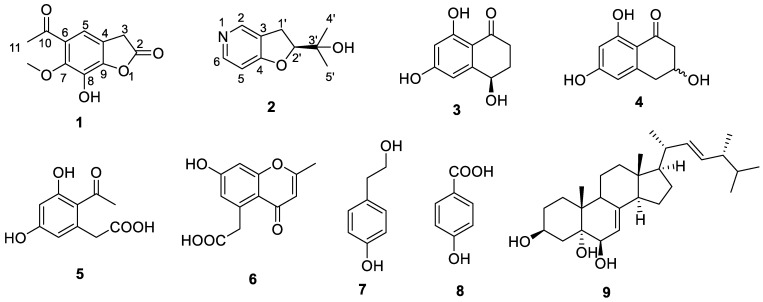
The structures of compounds **1**–**9**.

**Figure 2:**
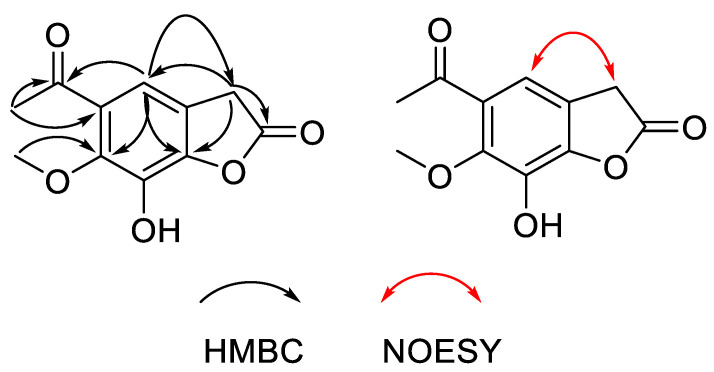
HMBC (single arrows) and NOESY (double arrows) correlations for compound **1**.

Compound **1**, obtained as a white powder, possessed the molecular formula C_11_H_10_O_5_ as assigned by the HRESIMS ion at *m*/*z* 223.0601 [M + H]^+^ (calculated for C_11_H_1__1_O_5_, 223.0601). The IR absorption bands at 3475 and 1651 cm^−1^ indicated hydroxy and carbonyl groups. By comparing the relevant NMR, MS and IR data with those of similar compounds in the literature [14], it was found that there was an additional carbonyl group in the structure of compound **1** (Figure 1). The ^13^C NMR spectrum of compound **1** displayed 11 carbon resonances, including seven quaternary C-atoms (*δ*_C_ 202.2, 172.5, 151.8, 150.2, 134.1, 129.4, 120.5), one aromatic or olefinic methine (*δ*_C_ 111.0), one methylene (*δ*_C_ 38.7), one methyl (*δ*_C_ 31.9) and one oxygenated methyl (*δ*_C_ 60.0) (Table 1). The ^1^H, DEPT135 and HSQC NMR data of compound **1** revealed the presence of one methyl group (*δ*_H_ 2.39, s) and one oxygenated methyl group (*δ*_H_ 3.69, s), one methylene group (*δ*_H_ 3.45, s), one aromatic or olefinic proton (*δ*_H_ 6.24, s) and one exchangeable proton (*δ*_H_ 9.51, br s) (Table 1). The planar structure of compound **1** was established by a comprehensive analysis of the 2D NMR spectroscopic data. The HMBC correlations (Figure 2) from H-11 (*δ*_H_ 2.39) to C-10 /C-6, from H-5 (*δ*_H_ 6.24) to C-10/C-3/C-7/C-9, from H_2_-3 (*δ*_H_ 3.45) to C-2/C-5/C-9 and from 7-OCH_3_(*δ*_H_ 3.69) to C-7, were observed. The aromatic proton H-5 also showed NOE correlations (Figure 2) with the methylene proton groups H_2_-3 in the NOESY spectrum. Therefore, the structure for compound **1** was determined as shown in Figure 1, and it was identified as a new compound named 5-acetyl-7-hydroxy-6-methoxybenzofuran-2(3*H*)-one.

**Figure 3:**
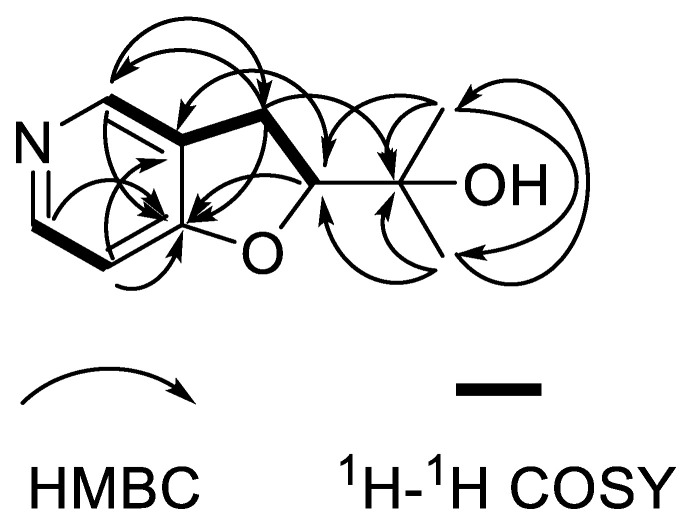
HMBC (single arrows) and ^1^H-^1^H COSY (bold lines) correlations for compound **2**.

**Figure 4:**
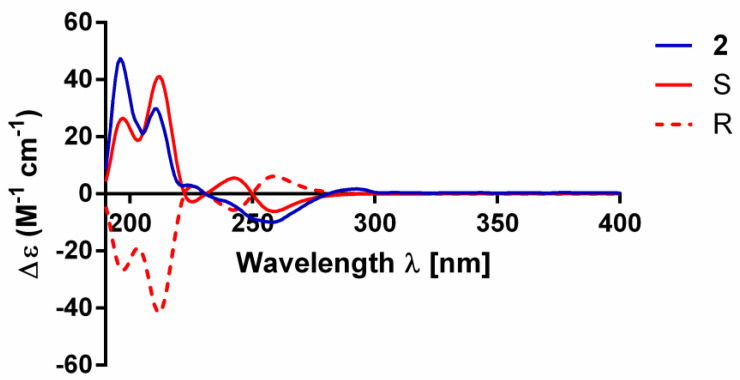
Experimental and calculated electronic circular dichroism (ECD) spectra of compound **2**.

Compound **2** was isolated as a white powder with the molecular formula of C_10_H_13_NO_2_ as derived from the HRESIMS ion at *m*/*z* 180.1017 [M + H]^+^ (calculated for C_10_H_14_NO_2_, 180.1019). The IR absorption bands at 3437 and 1026 cm^−1^ indicated hydroxy and carbon-nitrogen bonds. The ^1^H NMR spectrum of compound **2** revealed the presence of two methyl protons (*δ*_H_ 1.25 and 1.22), two ortho-coupled aromatic protons (*δ*_H_ 6.69, d, *J* = 8.2 Hz and 7.77, d, *J* = 7.7 Hz), one aromatic proton (*δ*_H_ 7.79, s), one methylene (*δ*_H_ 3.17, dd, *J* = 8.9, 4.5 Hz) and one oxygenated aliphatic methine (*δ*_H_ 4.63, t, *J* = 8.8 Hz) (Table 2). The ^13^C NMR spectrum display 10 carbon signals, including three quaternary C-atoms (*δ*_C_ 163.8, 128.2, 72.5), two methyls (*δ*_C_ 25.1, 25.3), one methylene (*δ*_C_ 31.1) and four methines (*δ*_C_ 131.4, 127.5, 108.9, 91.1) (Table 2). Analysis of the hydrogen and carbon quantities obtained from the above NMR data and the mass spectrometric data, found that there was a hydroxyl group in the structure of the compound. The planar structure of compound **2** was established by a comprehensive analysis of the 2D NMR spectral data. The ^1^H-^1^H COSY correlations (Figure 3) of H-2′/H-1′, H-6/H-5 and H-1′/H-2, could be observed. The HMBC correlations (Figure 3) from H-6 (*δ*_H_ 7.77) to C-4, from H-5(*δ*_H_ 6.69) to C-3/C-4, from H-2 (*δ*_H_ 7.79) to C-1′/C-4, from H-1′ (*δ*_H_ 3.17) to C-2′/C-3/C-2/C-4/C-3′, fromH-2′ (*δ*_H_ 4.63) to C-4/C-3, from H-4′ (*δ*_H_ 1.25) to C-2′/C-3′/C-5′ and from H-5′ (*δ*_H_ 1.22) to C-2′/C-3′/C-4′ also can be observed in the HMBC spectrum of compound **2**. The absolute configuration of compound **2** was determined by comparing the experimental and calculated ECD spectra using a time-dependent density-functional theory (TDDFT). As a result, the calculated ECD spectrum of compound **2** matched well with the experimental spectrum (Figure 4), which indicated the absolute configuration to 2’*S*. Thus, the complete structure of compound **2** was established as shown in Figure 1.

Based on the comparison of their NMR and MS data with those reported in the literature, the structures of compounds **3**–**9** were identified as 4,6,8-trihydroxy- 3,4-dihydronaphthalen-1(2*H*)-one (**3**) [15], 3,6,8-trihydroxy-3,4-dihydronaphthalen-1(2*H*) -one (**4**) [16,17], curvulin acid (**5**) [18,19], 2-methyl-5-carboxymethyl-7-hydroxychromone (**6**) [20], tyrosol (**7**) [21,22], *p*-hydroxybenzoic acid (**8**) [23] and cerevisterol (**9**) [24].

All of the isolated compounds were evaluated for their phytotoxicity against the fresh tender leaves of *Hevea brasiliensis*. The results show that none of them were phytotoxic. Additionally, the compounds were subjected to antimicrobial assay against three bacteria (*E. coli*, *Methicillin-resistant Staphylococcus aureus* and *Micrococcus luteus*). However, no compounds showed antimicrobial activity.

## 3. Experimental Section

### 3.1. General Experimental Procedures

Column chromatography (CC) was conducted on silica gel (200~300 μm, 60~80 μm) (Qingdao Marine Chemical Factory, Qingdao, China), Sephadex LH-20 (Cytiva, Uppsala, Sweden) and ODS reverse phase silica gel (40–60 µm; Osaka Soda Co., Ltd., Hyogo, Japan). The HPLC was performed on a Waters 1525 HPLC with an analytical column (Waters XBridge C18, 150 mm × 2.1 mm, 3.5 μm) or a semi-preparative column (Waters XBridge C18, 250 mm × 10 mm, 5 μm) (Waters Corporation, Milford, MA, USA). NMR experiments were performed on a Bruker AVANCE 800 MHz or 400 MHz NMR spectrometer (Bruker Corporation, Karlsruhe, Germany) with TMS (tetramethylsilane) as the internal standard (*δ* in ppm, *J* in Hz); HRESIMS were measured on an Agilent 6210 TOF LC-MS spectrometer (Agilent Technologies Inc., Palo Alto, CA, USA). UV and IR data were measured on a UV-2550 spectrometer (Shimadzu, Japan) and a Nicolet 380 Infrared Spectrometer (Thermo Fisher, Waltham, MA, USA), respectively, (Supplementary Materials).

### 3.2. Phytopathogenic Fungal Material

The fungal strain *C. cassiicola* CC01 (CGMCC 3.20258) was isolated from rubber trees collected from the plantations in Yangjiang, Guangdong province. The strain was maintained in our laboratory. The strain of *C. cassiicola* CC01 was grown on potato glucose medium (potato 200.0 g, glucose 20.0 g, agar 18.0 g, pure water 1.0 L, sterilized at 121 °C for 20 min in a high-pressure steam sterilization pot) and cultured at 28 °C for 4 days. After that, the activated strain was inoculated into a 500 mL flask containing 200 mL PDB liquid medium (potato 200.0 g, glucose 20.0 g, pure water 1.0 L, sterilized at 121 °C for 20 min in a high-pressure steam sterilization pot) and incubated at a constant temperature shaker for 72 h (160 r/min, 28 °C). Then 10 mL of the liquid seed solution was inoculated into a solid medium (rice 40.0 g, 60 mL water, sterilized at 121 °C for 20 min in a high-pressure steam sterilization pot) for fermentation for 30 days.

### 3.3. Extraction and Isolation

The strain *C. cassiicola* CC01 was extracted three times with an equal volume of ethyl acetate and the organic solvents were combined and evaporated under reduced pressure to produce a crude extract (83.08 g). Then the extract was separated onto a silica gel CC with CH_2_Cl_2_/MeOH (100:0 to 0:100, *v*/*v*) to obtain nine fractions (Fr.A–Fr.H). Fr.G, was subjected to an ODS reverse-phase CC under reduced pressure (MeOH-H_2_O, 20% to 80%, *v*/*v*), Sephadex LH-20 CC and semi-preparative HPLC to yield compound **1** (30.7 mg, t*_R_* = 8.1 min; 254 nm, 3.0 mL/min, 20% MeOH in H_2_O) and compound **6** (5.9 mg, t*_R_* = 15.4 min; 280 nm, 3.0 mL/min, 40% MeOH in H_2_O). Fr.E was separated by ODS reverse-phase CC under reduced pressure (MeOH-H_2_O, 20% to 80%, *v*/*v*), Sephadex LH-20 CC and semi-preparative HPLC (280 nm, 3.0 mL/min, 30% to 65% MeOH in H_2_O) to obtain compound **2** (5 mg, t*_R_* = 21.7 min), compound **3** (2.4 mg, t*_R_* = 14.1 min) and compound **7** (2.7 mg, t*_R_* = 13.8 min). Fr.H1 and Fr.F2 subfractions were separated by ODS reverse-phase CC under reduced pressure (MeOH-H_2_O, 20% to 80%, *v*/*v*), Sephadex LH-20 CC and semi-preparative HPLC to obtain compound **5** (3.1 mg, t*_R_* = 7.7 min; 254 nm, 3.0 mL/min, 40% MeOH in H_2_O), compound **4** (2.4 mg, t*_R_* = 12.4 min; 280 nm, 3.0 mL/min, 25% MeOH in H_2_O), compound **8** (52.1 mg, t*_R_* = 23.5 min; 280 nm, 3.0 mL/min, 45% MeOH in H_2_O) and compound **9** (20 mg, t*_R_* = 18.1 min; 280 nm, 3.0 mL/min, 45% MeOH in H_2_O).

### 3.4. Spectral and Physical Data of Compounds **1** and **2**

Compound **1**: white powder; IR ν_max_ 3475, 3303, 2987, 1651, 1391 cm^−^^1^; UV(MeOH)*λ*_max_ (log *ε*) 254 (2.21), 284 (2.50), 340 (1.58) nm; ^1^H (400 MHz, DMSO-*d*_6_) and ^13^C (100 MHz, DMSO-*d*_6_) NMR data, see Table 1; HRESIMS: *m*/*z* 223.0601 [M + H]^+^ (calcd for C_11_H_10_O_5_, 223.0601).

Compound **2**: white powder; [α]^20^_D_ 204.3 (*c* 0.1, MeOH); IR ν_max_ 3437, 2925, 1551, 1387, 1026 cm*^−^*^1^; UV(MeOH)*λ*_max_ (log *ε*) 223 (1.97), 261 (2.19), 295 (1.76) nm; ECD (MeOH) λ_max_ (∆ε) 210 (+5.34), 229 (+0.20), 250 (−1.56), 264 (−1.40), 291 (+0.30) nm; ^1^H (800 MHz, CD_3_OD) and ^13^C (200 MHz, CD_3_OD) NMR data, see Table 2; HRESIMS: *m*/*z* 180.1017 [M + H]^+^ (calcd for C_10_H_13_NO_2_, 180.1019).

### 3.5. Electronic Circular Dichroism Calculation

Monte Carlo conformational searches were conducted by means of the Spartan’s 14 software using Merck Molecular Force Field (MMFF). The conformers with Boltzmann-population of over 5% were chosen for ECD calculations, and then the conformers were initially optimized at B3LYP/6-31g level in gas. The theoretical calculation of ECD was carried out in MeOH using a Time-dependent Density functional theory (TD-DFT) at the B3LYP/6-31+g (d, p) level for all conformers of compound **2**. Rotatory strengths for a total of 30 excited states were calculated. ECD spectra were generated using the program SpecDis 1.6 (University of Würzburg, Würzburg, Germany) and GraphPad Prism 5 (University of California San Diego, USA) from dipole-length rotational strengths by applying Gaussian band shapes with sigma = 0.3 eV.

### 3.6. Phytotoxic Assay

The activities of the isolated compounds were measured by leaf puncture [25] to test their phytotoxicity. Healthy and fresh rubber tree leaves in the light green stage (high-resistance germplasm of Reyan7-33-97 planted in the Yanfeng Experimental Base of the Chinese Academy of Tropical Agricultural Sciences) were used as inoculum material. The compounds were tested at 0.1 mg/mL and 1 mg/mL (dissolved in 5% methanol-aqueous solution) on rubber tree leaves, using a small insect needle (over 1 mm^2^). One drop of the test sample (5 μL) was placed on the gently punctured spot, with 10 min vacuum infiltration. Leaflets were maintained in a moist environment at 28 °C under alternate light (photoperiod 12 h/12 h) and the effects of the metabolites on the leaves were observed at 72 h.

### 3.7. Antimicrobial Assay

The antimicrobial activities of the metabolites were measured by the filter paper bacteriostatic method. Compounds **1**–**9** were evaluated against three bacteria (*E. coli*, *Methicillin-resistant Staphylococcus aureus* and *Micrococcus luteus*). Each compound was tested at 1.0 mg/mL in a DMSO solvent. DMSO solution was used as a negative control and kanamycin solution (10 mg/mL) was used as a positive control. Each test was treated with three replicates and incubated at 28 °C for 12 h. Then the size of the inhibition zone was recorded.

## 4. Conclusions

In this study, nine compounds were isolated and purified from the fermentation extract of *C.* *cassiicola* CC01, an important phytopathogenic fungus causing rubber leaf diseases. Compounds **1** and **2** were identified as new compounds and compounds **3**–**9** were discovered from this phytopathogenic fungus for the first time. Compound **3** was previously identified from a freshwater fungus, YMF 1.01029 and showed a weak nematocidal activity against xylocysts. Compound **4** is a novel metabolite with antifungal activity isolated from *Phomopsis* sp. [26]. Compound **5** was reported to have a weak anti-HIV-1 integrase chain transfer activity, with an IC_50_ value of 75.1 μmol/L Compound **6** is a chromone derivative firstly isolated from *Rhubarb equinosa* in 1984 [27]. Compound **7** can be used for the synthesis of medolol, betalol, salidroside and other drugs [28]. Compound **9** was first isolated from *Myriapora truncate* in nature in 1985 [29]. All of the isolated compounds were evaluated for their phytotoxicity, and the results show that none of them were phytotoxic at 0.1 mg/mL and 1 mg/mL, indicating that there may be some trace phytotoxic components of this fungus that have not been isolated. The subsequent exploration for phytotoxic metabolites will continue. Although no pathogenic molecules have been found from this phytopathogenic fungus, the new compounds **1** and **2** and the other seven natural products were discovered from the secondary metabolites of this fungus for the first time, which further enriched the chemical diversity produced by this pathogenic fungus.

## Figures and Tables

**Table 1:** ^1^H (400 MHz) and ^13^C (100 MHz) NMR data for compound **1** (DMSO-*d*_6_).

Position	*δ*_H_ (*J* in Hz)	*δ*_C_, Type
2		172.5, C
3	3.45, s	38.7, CH_2_
4		129.4, C
5	6.24, s	111.0, CH
6		120.5, C
7		134.1, C
8		150.2, C
8-OH	9.51, br s	
9		151.8, C
10		202.2, C
11	2.39, s	31.9, CH_3_
7-OCH_3_	3.69, s	60.0, CH_3_

**Table 2:** ^1^H (800 MHz) and ^13^C (200 MHz) NMR data for compound **2** (methanol-*d*_4_).

Position	*δ*_H_ (*J* in Hz)	*δ*_C_, Type
2	7.79, s	127.5, CH
3		128.2, C
4		163.8, C
5	6.69, d (8.2)	108.9, CH
6	7.77, d (7.7)	131.4, CH
1′	3.17, dd (8.9, 4.5)	31.1, CH_2_
2′	4.63, t (8.8)	91.1, CH
3′		72.5, C
4′	1.25, s	25.1, CH_3_
5′	1.22, s	25.3, CH_3_

## Data Availability

Not available.

## Supplementary Materials

The following supporting information can be downloaded at https://www.mdpi.com/article/10.3390/molecules27217360/s1, Figure S1–S7: ^1^H, ^13^C, DEPT135, HSQC, HMBC, NOESY and HRESIMS spectra of compound **1**; Figure S8–S13: ^1^H, ^13^C, HSQC, HMBC, ^1^H-^1^H COSY and HRESIMS spectra of compound **2**.

## Sample Availability

Samples of the compounds are available from the authors.
