# Supplementary material for: Isolation and Identification of Secondary Metabolites Produced by Phytopathogenic Fungus Corynespora cassiicola from Hevea brasiliensis
Source: Molecules. 2022 Oct 29;27(21):7360. doi: 10.3390/molecules27217360 (PMC9657256; doi:10.3390/molecules27217360)

Figure S1. ^{1}H NMR (400 MHz, DMSO-d_6) spectrum of new compound **1**

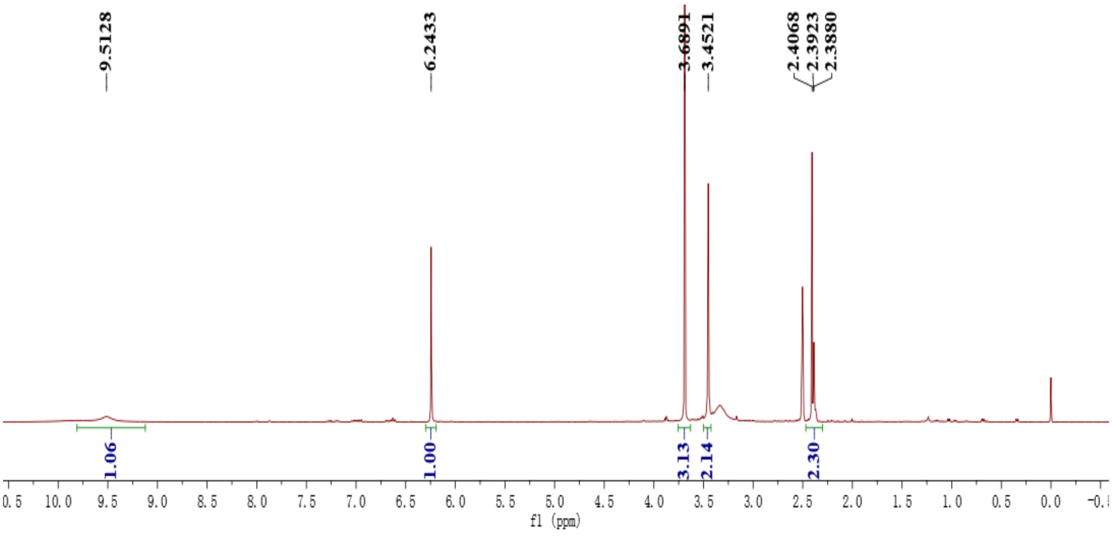

Figure S2. ^{13}C NMR (100 MHz, DMSO-d_6) spectrum of new compound **1**

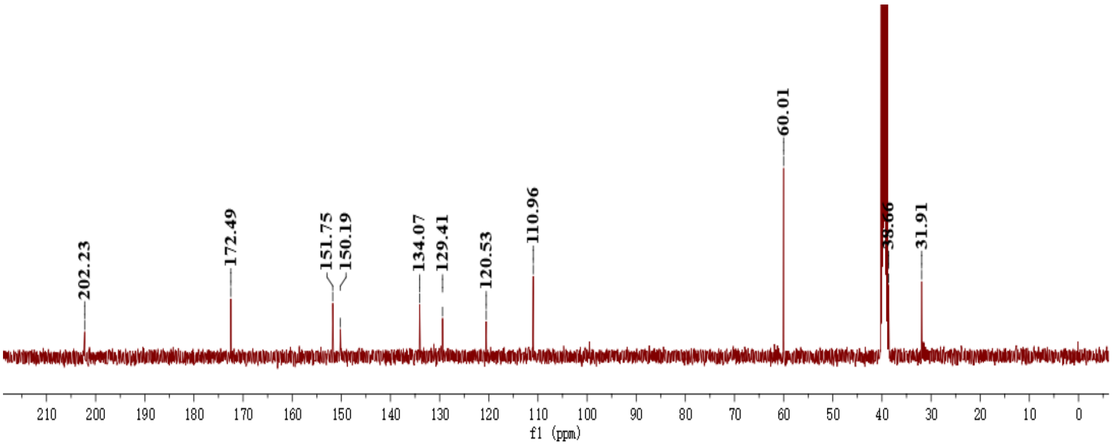

Figure S3. DEPT135 spectrum of new compound **1**

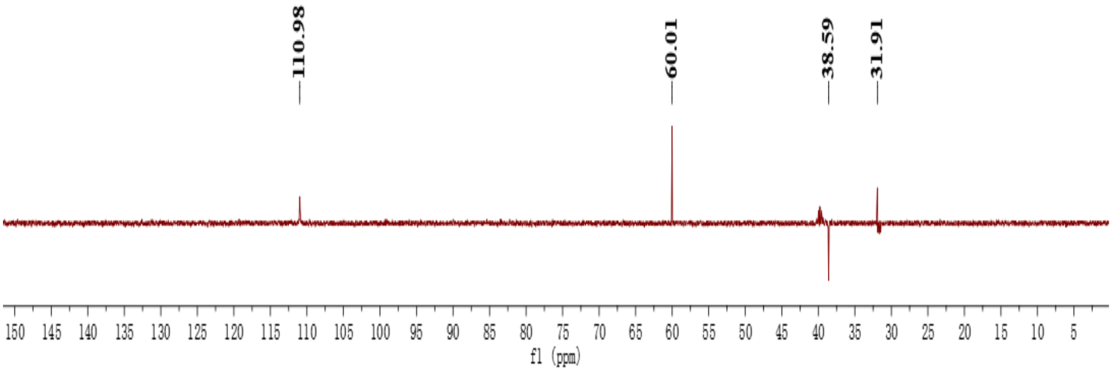

Figure S4. HSQC spectrum of new compound **1**

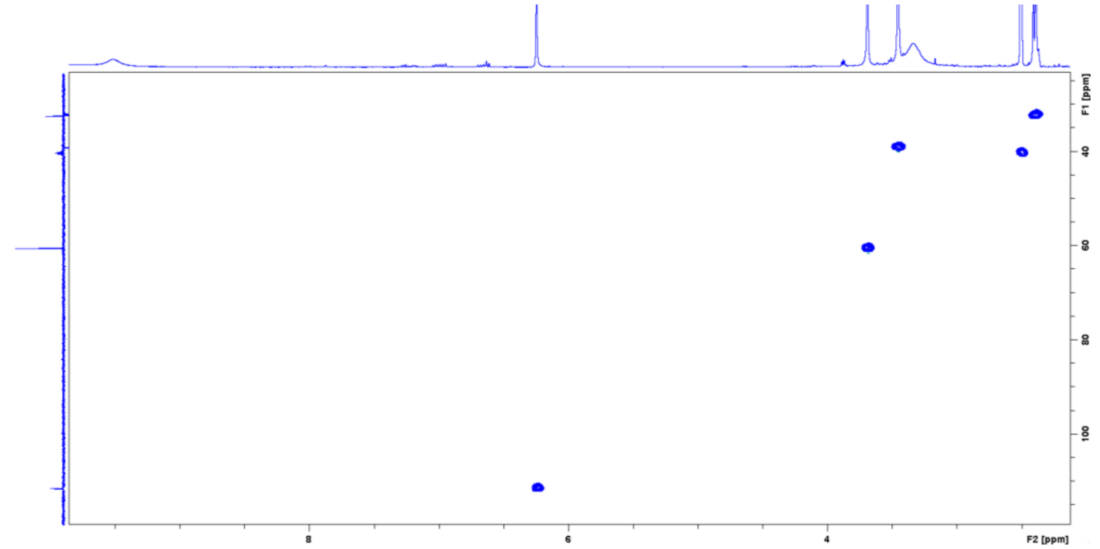

Figure S5. HMBC spectrum of new compound **1**

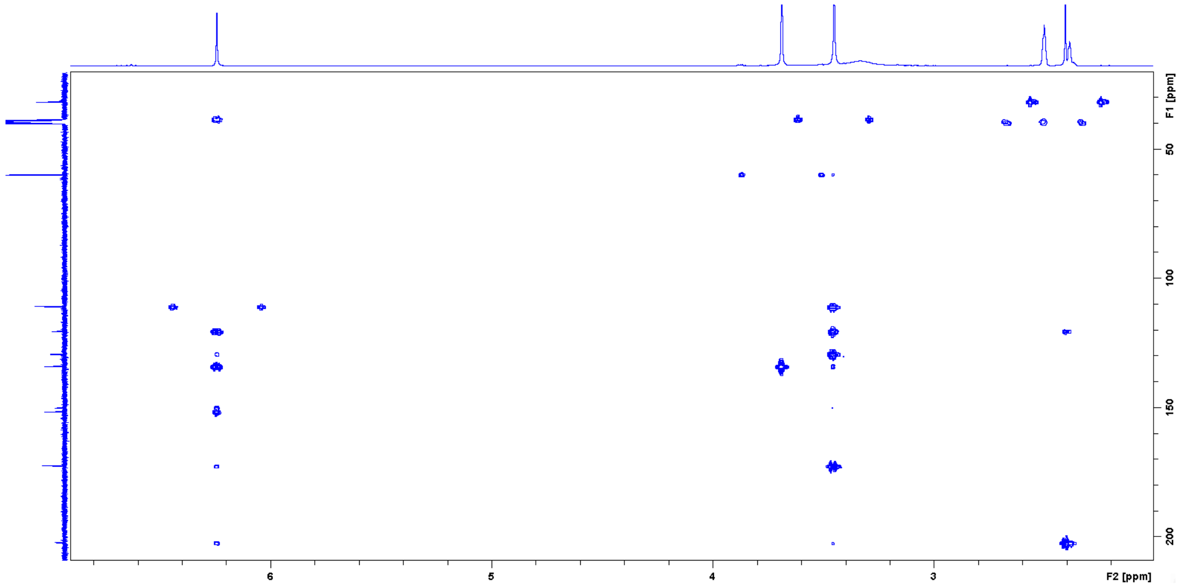

Figure S6. NOESY spectrum of new compound **1**

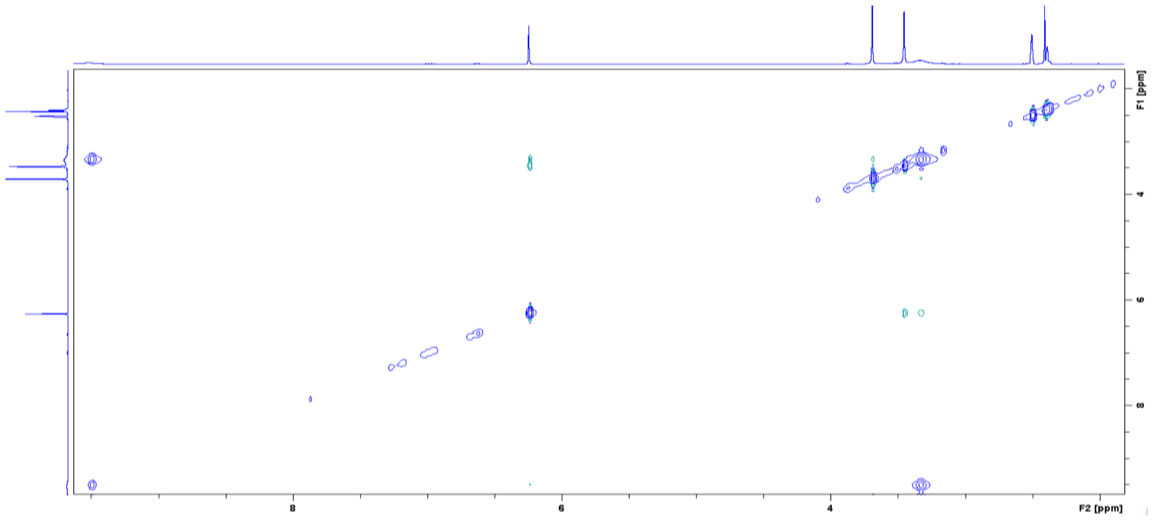

Figure S7. The HRESIMS spectrum of the new compound **1**

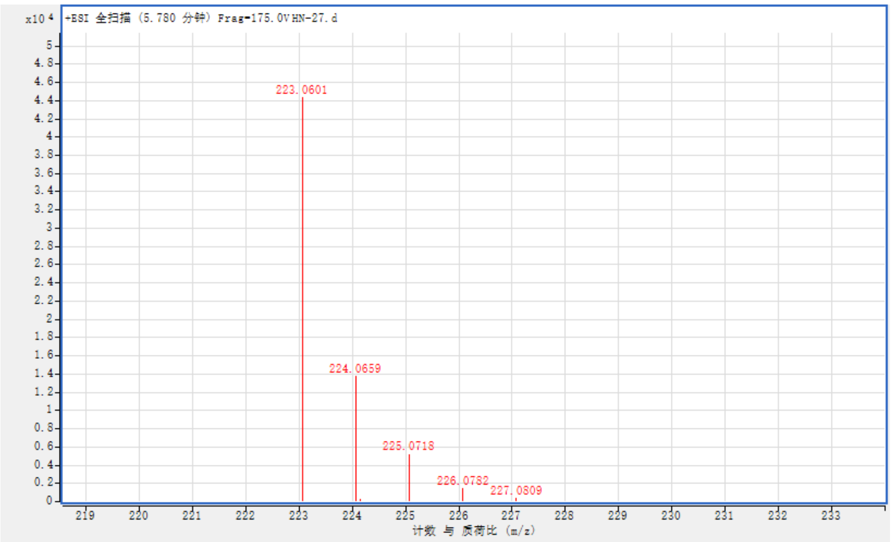

Figure S8. ^{1}H NMR (800 MHz, CD_3OD) spectrum of new compound **2**

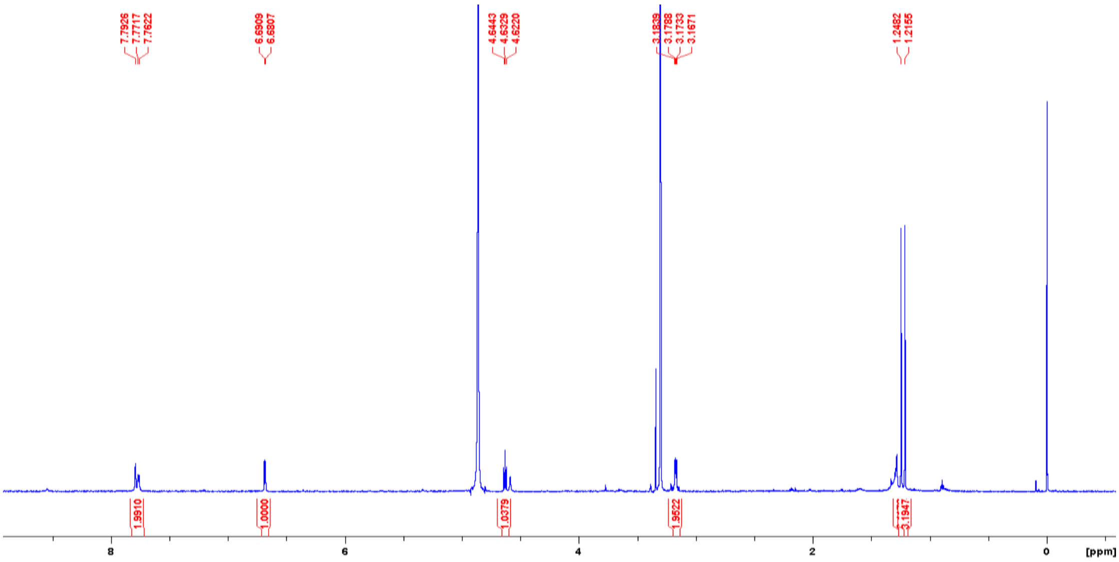

Figure S9. ^{13}C NMR (200 MHz, CD_3OD) spectrum of new compound **2**

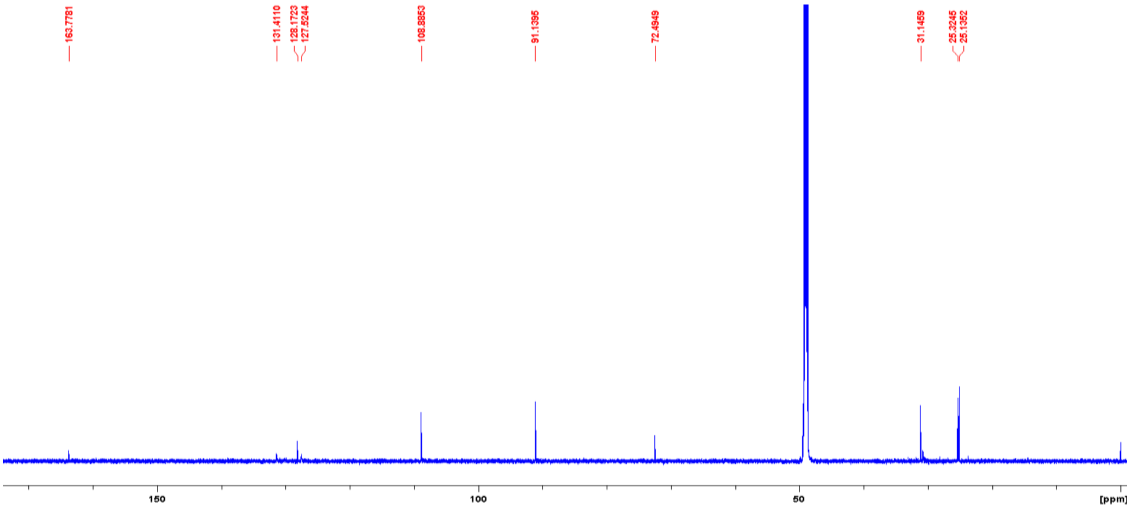

Figure S10. HSQC spectrum of new compound **2**

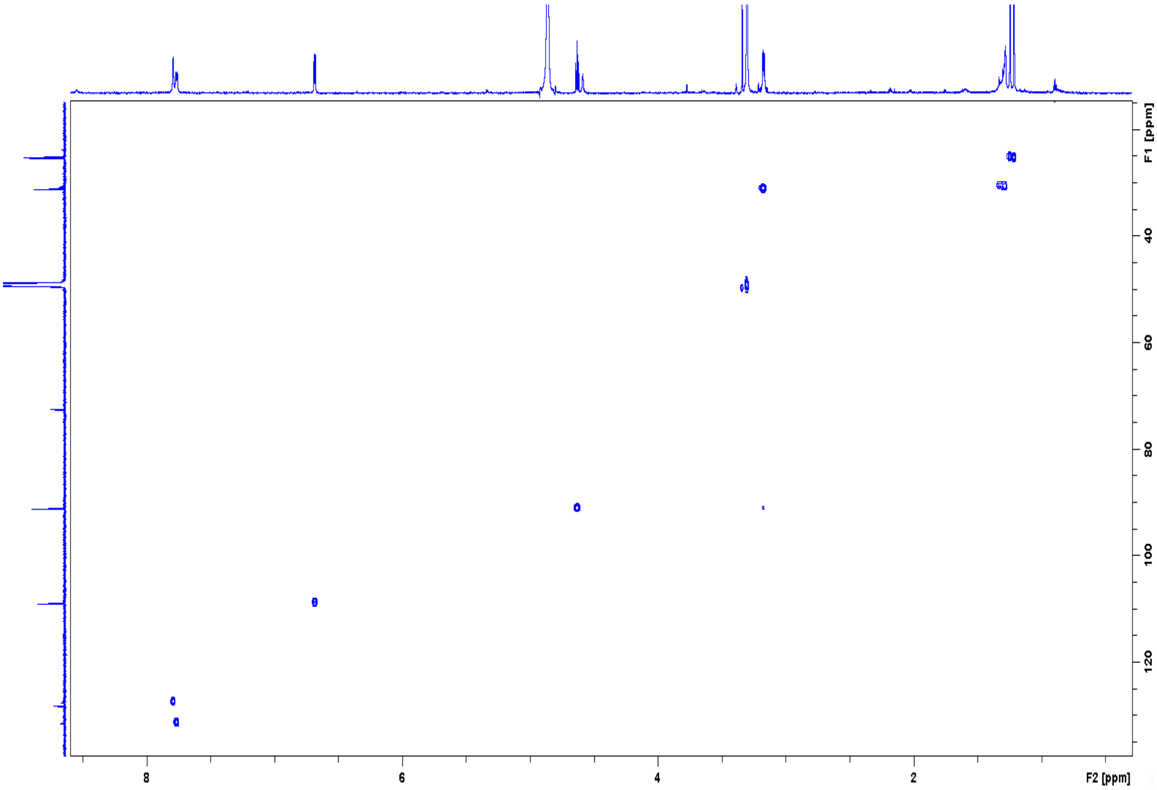

Figure S11. HMBC spectrum of new compound **2**

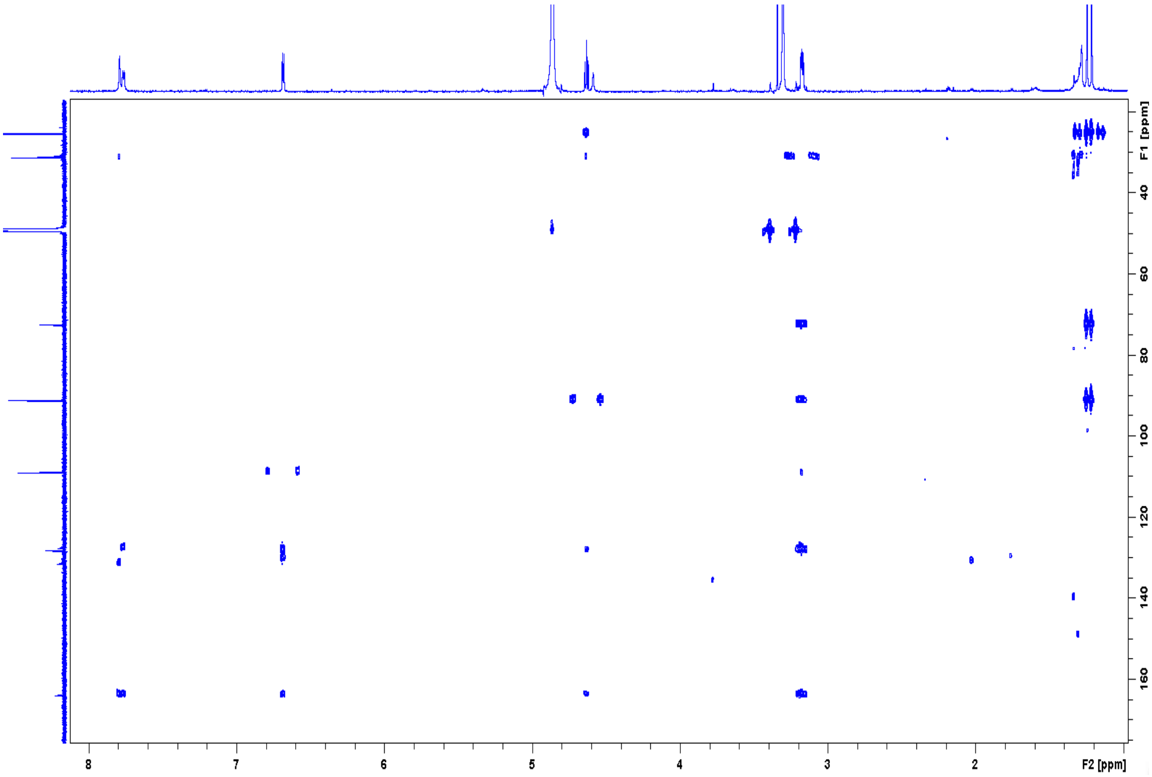

Figure S12. ^{1}H-^{1}H COSY spectrum of new compound **2**

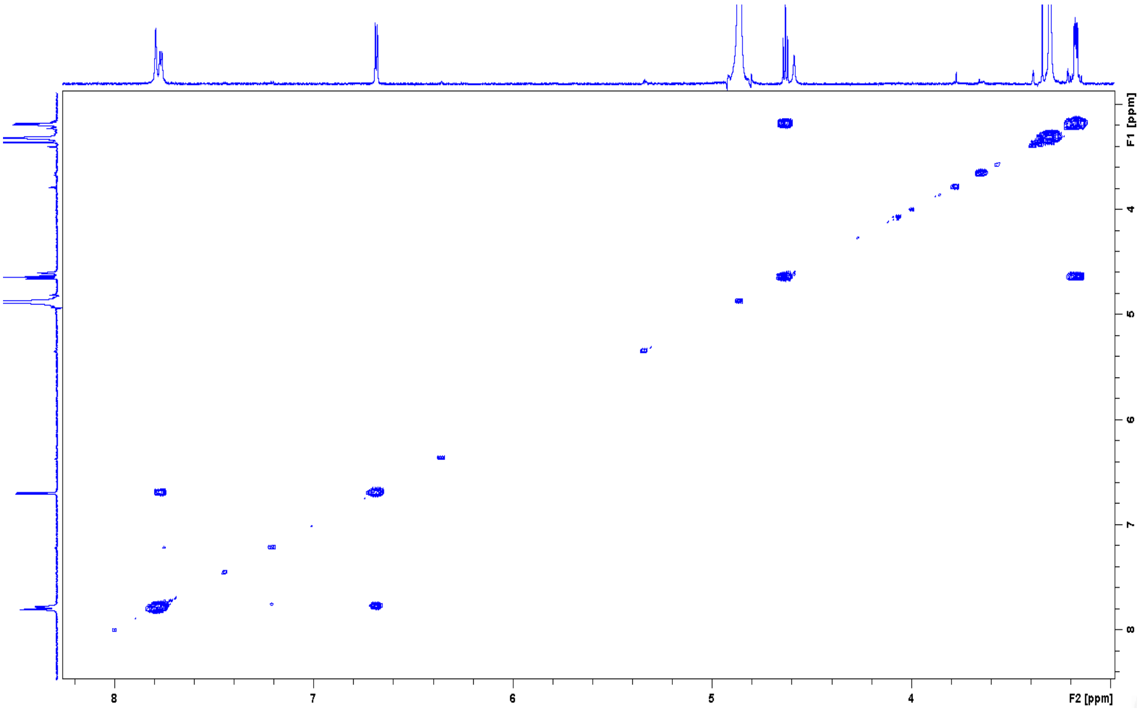

Figure S13. The HRESIMS spectrum of new compound **2**

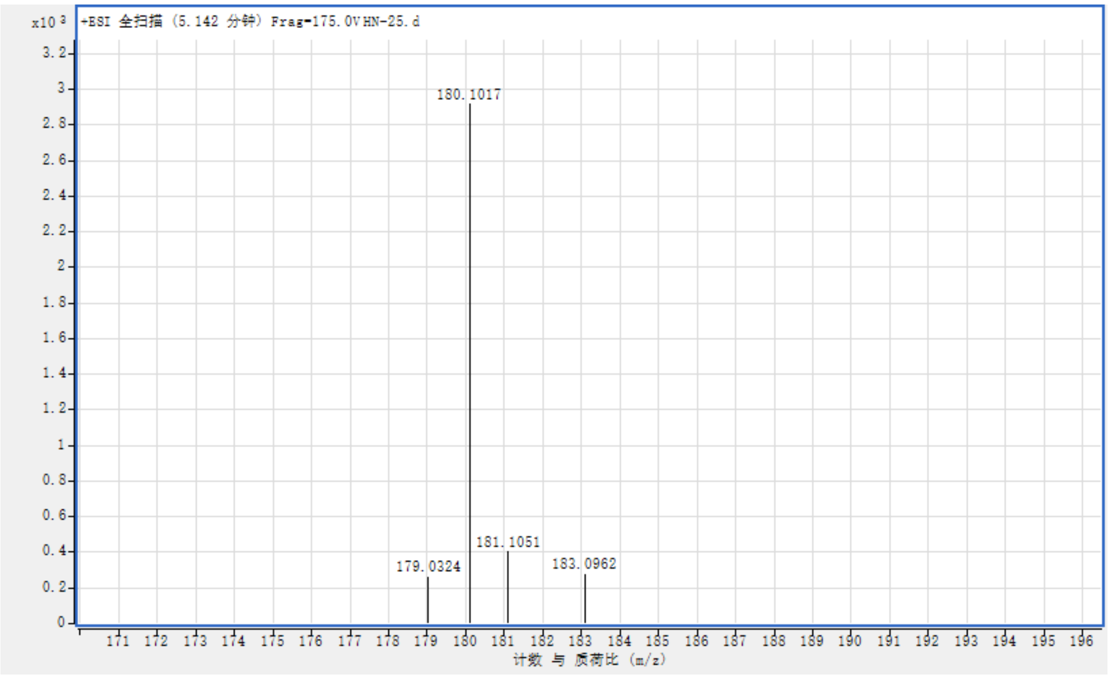

Supplement: Supplementary file 1 [file molecules-27-07360-s001.zip › molecules-1985554-supplementary.pdf]
